# Microfluidic/HPLC combination to study carnosine protective activity on challenged human microglia: Focus on oxidative stress and energy metabolism

**DOI:** 10.3389/fphar.2023.1161794

**Published:** 2023-03-29

**Authors:** Anna Privitera, Vincenzo Cardaci, Dhanushka Weerasekara, Miriam Wissam Saab, Lidia Diolosà, Annamaria Fidilio, Renaud Blaise Jolivet, Giuseppe Lazzarino, Angela Maria Amorini, Massimo Camarda, Susan Marie Lunte, Filippo Caraci, Giuseppe Caruso

**Affiliations:** ^1^ Department of Drug and Health Sciences, University of Catania, Catania, Italy; ^2^ Department of Biomedical and Biotechnological Sciences, University of Catania, Catania, Italy; ^3^ Vita-Salute San Raffaele University, Milano, Italy; ^4^ Scuola Superiore di Catania, University of Catania, Catania, Italy; ^5^ Ralph N. Adams Institute for Bioanalytical Chemistry, University of Kansas, Lawrence, KS, United States; ^6^ Department of Pharmaceutical Chemistry, University of Kansas, Lawrence, KS, United States; ^7^ Unit of Neuropharmacology and Translational Neurosciences, Oasi Research Institute-IRCCS, Troina, Italy; ^8^ Maastricht Centre for Systems Biology (MaCSBio), Maastricht University, Maastricht, Netherlands; ^9^ STLab Srl, Catania, Italy; ^10^ Department of Chemistry, University of Kansas, Lawrence, KS, United States

**Keywords:** carnosine, human microglia, inflammation, oxidative stress, energy metabolism, depression, microfluidics, HPLC

## Abstract

Carnosine (β-alanyl-L-histidine) is a naturally occurring endogenous peptide widely distributed in excitable tissues such as the brain. This dipeptide possesses well-demonstrated antioxidant, anti-inflammatory, and anti-aggregation properties, and it may be useful for treatment of pathologies characterized by oxidative stress and energy unbalance such as depression and Alzheimer’s disease (AD). Microglia, the brain-resident macrophages, are involved in different physiological brain activities such synaptic plasticity and neurogenesis, but their dysregulation has been linked to the pathogenesis of numerous diseases. In AD brain, the activation of microglia towards a pro-oxidant and pro-inflammatory phenotype has found in an early phase of cognitive decline, reason why new pharmacological targets related to microglia activation are of great importance to develop innovative therapeutic strategies. In particular, microglia represent a common model of lipopolysaccharides (LPS)-induced activation to identify novel pharmacological targets for depression and AD and numerous studies have linked the impairment of energy metabolism, including ATP dyshomeostasis, to the onset of depressive episodes. In the present study, we first investigated the toxic potential of LPS + ATP in the absence or presence of carnosine. Our studies were carried out on human microglia (HMC3 cell line) in which LPS + ATP combination has shown the ability to promote cell death, oxidative stress, and inflammation. Additionally, to shed more light on the molecular mechanisms underlying the protective effect of carnosine, its ability to modulate reactive oxygen species production and the variation of parameters representative of cellular energy metabolism was evaluated by microchip electrophoresis coupled to laser-induced fluorescence and high performance liquid chromatography, respectively. In our experimental conditions, carnosine prevented LPS + ATP-induced cell death and oxidative stress, also completely restoring basal energy metabolism in human HMC3 microglia. Our results suggest a therapeutic potential of carnosine as a new pharmacological tool in the context of multifactorial disorders characterize by neuroinflammatory phenomena including depression and AD.

## 1 Introduction

Carnosine is a natural occurring dipeptide (beta-alanyl-L-histidine) and an over-the-counter food supplement (Gulewitsch and Amiradžibi, 1900). This dipeptide is synthetized starting from β-alanine and L-histidine *via* the activity of the carnosine synthase 1 enzyme, while its degradation into its constituting amino acids depends on cytosolic and plasmatic carnosinases (Kalyankar and Meister, 1959; Winnick and Winnick, 1959). Carnosine can be found in several mammalian tissues, with the highest tissue concentrations observed in the brain (up to 5 mM) as well as in skeletal and cardiac muscle (up to 20 mM) (Hipkiss et al., 1998; Gariballa and Sinclair, 2000).

There are different pre-clinical studies showing the ability of carnosine to exert a neuroprotective and anti-inflammatory activity (Caruso et al., 2019a) through a multimodal mechanism of action that includes the scavenging of free radicals (Chen et al., 2009; Prokopieva et al., 2016), the downregulation of pro-inflammatory markers (Kubota et al., 2020), as well as the modulation of immune cells such as macrophages and microglia (Caruso et al., 2019b), regulating their production of reactive oxygen and nitrogen species (ROS and RNS, respectively) and polarization (Caruso et al., 2017a; Fresta et al., 2018). Carnosine has also shown to improve global cognitive function in the elderly (Rokicki et al., 2015) and its therapeutic potential has been considered for the treatment of different neuropsychiatric disorders such as Parkinson’s disease (PD), schizophrenia, Alzheimer’s disease (AD), attention-deficit/hyperactivity disorder, and cognitive impairment in the elderly (Boldyrev et al., 2008; Chengappa et al., 2012; Ghajar et al., 2018; Caruso et al., 2021; Caruso et al., 2022b).

Microglia represent the immune cells of the central nervous system (CNS) and play a key role in brain development, memory, synaptic plasticity, and neurogenesis (Yirmiya et al., 2015). Microglial dyshomeostasis and/or hyperactivation due to pro-inflammatory/pro-oxidant conditions (e.g., lipopolysaccharides (LPS) infections) or aging characterized by microglial decline and senescence can contribute to the pathogenesis of major depression and associated impairments in neuroplasticity and neurogenesis (Delpech et al., 2015). Numerous studies have shown that activated microglia initiate an inflammatory process in various neurogenerative disorders including AD, PD, and multiple sclerosis (Liu and Hong, 2003; Glass et al., 2010; Stephenson et al., 2018; Caruso et al., 2022a). The activation of microglia has also been associated to a phenomenon known as “oxidative stress” (Caruso et al., 2019d), defined as an excessive production of ROS/RNS that overcomes the antioxidant defense system (Gunasekara et al., 2014; Weidinger and Kozlov, 2015). High levels of ROS/RNS have been related to the development of numerous pathologies including cancer, atherosclerosis, cardiovascular disease, diabetes, PD, and AD.

LPS, expressed in the external membrane of Gram-negative bacteria, represents one of the main inflammatory inducers of microglial cells (Cui et al., 2012). LPS-induced microglial activation triggers neuroinflammatory processes through the secretion of different types of cytokines and eicosanoids (Lykhmus et al., 2016). LPS-activated signaling pathways have been shown to significantly increase the production of pro-inflammatory markers such as nitric oxide (NO) and ROS, tumor necrosis factor-alpha (TNF-α), interleukin-6 (IL-6), and prostaglandin E2 (PGE2) (Hanada and Yoshimura, 2002; Bellezza et al., 2013). Furthermore, microglial cells represent a common model of LPS-induced activation to identify novel pharmacological targets for depression and AD and develop potential therapeutic approaches able to attenuate the production and release of pro-inflammatory mediators (Park et al., 2007).

Recent pre-clinical and clinical studies have shown that the response to bacterial infection is characterized by enhanced levels of circulating C-reactive protein (Iraz et al., 2015; Engler et al., 2017; Kyvelidou et al., 2018). It has also been observed that the administration of LPS in animals leads to a behavioral phenotype mimicking anhedonia and affective symptoms currently detectable in depressed patients (Lasselin et al., 2020). Furthermore, significant associations between blood concentrations of LPS-induced pro-inflammatory cytokines and affective and cognitive symptoms have been observed in depressed patients (Suarez et al., 2003). Many research studies have also linked the impairment of energy metabolism to the onset of depressive episodes (Gu et al., 2021); in fact, abnormal energy metabolism represents one of the key mechanisms for the occurrence and development of this disease. Of note, systemic inflammation has been associated to disruption of energy metabolism at CNS level (Kealy et al., 2020). In particular, LPS-induced inflammation and IL-1β can trigger hypoglycemia and reduce glucose levels in the brain (Kealy et al., 2020). All these molecular events related to LPS toxicity can contribute to the onset of cognitive symptoms in depression (Kotulla et al., 2018).

It is well-known that energy metabolism is linked to the production of ROS and fundamental enzymes part of the metabolic pathways can be affected by redox reactions. During the physiological aging as well as the onset and progression of numerous age-related diseases, such as atherosclerosis and neurodegenerative diseases, the interplay existing between energy metabolism and ROS becomes even more clear (Quijano et al., 2016; Trostchansky et al., 2016).

In the present study, we first investigated the toxic potential of LPS + ATP combination in the absence or presence of carnosine. We conducted these studies in human microglia (HMC3) in which this combination of stimuli has been shown to induce cell toxicity paralleled by pro-inflammatory phenomena (Li et al., 2018). Additionally, to elucidate the molecular mechanisms underlying the protective effect of carnosine, we evaluated the ability of this dipeptide to modulate ROS production as well as the variation of parameters representative of cellular energy metabolism and oxidative stress in activated HMC3 cells. In the present manuscript we show for the first time that carnosine is able to counteract microglia cell death induced by LPS + ATP by reducing oxidative stress and rescuing basal energy metabolism conditions.

## 2 Materials and methods

### 2.1 Materials and reagents

C-Chip disposable hemocytometers were obtained from Li StarFish S.r.l. (Naviglio, MI, Italy). HMC3 (human microglia) cells (ATCC^®^ CRL-3304™), fetal bovine serum (FBS), trypsin-EDTA solution, Eagle’s Minimum Essential Medium (EMEM), and penicillin/streptomycin solution were supplied by American Type Culture Collection (ATCC, Manassas, VA, United States). Centrifuge tubes equipped with 3 kDa molecular weight cut-off filters, methanol, water, chloroform, and far-UV acetonitrile were purchased from VWR International (West Chester, PA, United States). Sylgard 184 polydimethylsiloxane (PDMS) prepolymer and curing agent were obtained from Ellsworth Adhesives (Germantown, WI, United States). Kits to perform RNA extraction (RNeasy Mini Kit) and cDNA synthesis (QuantiTect Rev. Transcription Kit) along with QuantiTect SYBR Green PCR Kits and QuantiTect Primer Assays were purchased from Qiagen (Hilden, Germany). 384-well plates were supplied by Roche Molecular Systems Inc (Pleasanton, CA, United States). PCR tubes and LoBind Microcentrifuge Tubes PCR Clean were obtained from Eppendorf (Hamburg, Germany). HPLC-grade acetonitrile was obtained by VWR Chemicals (Briare, France). All water used in our study was Ultrapure (18.3 MΩ cm) (Milli-Q Synthesis A10, Millipore, Burlington, MA, United States). All the remaining materials, of analytical grade, were supplied by Sigma-Aldrich Corporate (St. Louis, MO, United States) or Thermo Fisher Scientific Inc. (Pittsburgh, PA, USA) unless specified otherwise.

### 2.2 Propagation and maintenance of cells

HMC3 cells were cultured in EMEM medium enriched with FBS (10%), streptomycin and penicillin (0.3 mg mL^–1^ and 50 IU mL^–1^, respectively), GlutaMAX (1 mM), sodium pyruvate (1 mM), and MEM non-essential amino acids by using 25 or 75 cm^2^ polystyrene culture flasks. Cells were maintained in a humidified environment (37°C, 95% air/5% CO_2_), and split every 3–5 days depending on cell confluence.

### 2.3 Analysis of cell status

The number of live and dead cells under our experimental conditions was determined by using a trypan blue exclusion assay as previously described (Fresta et al., 2018). Each cell suspension was diluted 1:1 to 1:3 (depending on cell density) with 0.4% trypan blue solution and loaded on a C-Chip disposable hemocytometer for cell status analysis. Live cells, characterized by intact cell membranes, excludes the trypan blue, while dead cells did not.

### 2.4 Intracellular ROS levels determination

On the day of the experiment, HMC3 cells were harvested by using trypsin/EDTA, counted, plated in 25 cm^2^ polystyrene culture flasks, and incubated in a humidified environment (95% air/5% CO_2_, 37°C) to allow the complete cell attachment. The day after, cells were left untreated (control) or treated with LPS (100 ng/mL for 24 h) plus ATP (5 mM for 30 min), in the absence or presence of carnosine (10 mM; 1 h pre-treatment). At the end of the stimulation, the intracellullar ROS levels in HMC3 cells were determined by using microchip electrophoresis with laser-induced fluorescence (ME-LIF) and 2′,7′-dichlorodihydrofluorescein diacetate (H_2_DCFDA) (Fresta et al., 2018) as previously described, with slight modifications. Briefly, in each 25 cm^2^ polystyrene culture flask the medium was removed, cells were washed with phosphate-buffered saline (PBS), and incubated with Dulbecco’s Modified Eagle Medium (DMEM) without phenol red-free containing H_2_DCFDA (10 μM) for 1 h (37°C, 95% air/5% CO_2_) (Fresta et al., 2018). Next, the medium was removed, cells were washed with PBS, harvested using trypsin/EDTA, centrifuged, and the obtained cell pellet was prepared and analyzed by ME-LIF (Caruso et al., 2017b). Specifically, cells were lysed with 50 μL of pure ethanol, the lysate solution was filtered with a 3 kDa molecular weight cut-off filter with centrifugation at 17,000 x *g* for s total of 10 min. Each filtered cell lysate (10 μL) was diluted 10 times by adding 90 μL of 10 mM boric acid, 2 mM β-cyclodextrin, and 3.5 mM sodium dodecyl sulfate at pH 9.2 obtaining a cell lysate solution with 10% ethanol. Twenty μL of this solution were run by using the PDMS-based microfluidic chips with a simple-T geometry, made by mixing PDMS prepolymer and curing agent in a 1:10 *w/w* ratio (Supplementary Figure S1). An aliquot of cell suspension coming from each flask was used for cell counting. The fabrication of each disposable hybrid PDMS-glass microchip used to perform ME-LIF analysis was based on a procedure described previously in details (Mainz et al., 2012; de Campos et al., 2015). Briefly, soft photolithography was selected to fabricate the SU-8 10 photoresist (Silicon, Inc., Boise, ID, United States) mold containing the microdevice design. The obtained wafer was soft baked through a programmable hotplate (Thermo Scientific, Asheville, NC, United States). Microchip channel designs were designed with the AutoCAD software (Autodesk Inc., San Rafael, CA, United States) and printed onto a transparency film (Infinite Graphics Inc., Minneapolis MN, United States). A transparency film mask was used to cover the coated wafer followed by UV light exposure (ABM Inc., San Jose, CA, United States), post-baking, developing, rinsing, and drying steps. The procedure ended with the wafer that was subjected to a hard bake. To obtain each disposable hybrid PDMS-glass microchip, the PDMS layer containing the embedded channels was sealed to a borofloat glass plate.

### 2.5 Analysis of metabolites

A well-established HPLC method was used for the analysis of the intracellular metabolites in deproteinized samples obtained by HMC3 cells under all our experimental conditions. At the end of the incubation, cells were pelleted, washed by using ice-cold PBS, and deproteinized by employing a protocol based on the use of organic solvents (e.g., acetonitrile) that allows the measurement of both acid labile and easily oxidizable compounds (Chen et al., 2009). Previously described ion pairing HPLC methods were used for the simultaneous separation of numerous metabolites including high-energy triphosphates, nicotinic coenzymes, reduced glutathione (GSH), nitrite and nitrate in the protein-free cell extracts (Romitelli et al., 2007; Lazzarino et al., 2018). Separation was obtained by using a Hypersil C-18, while the HPLC apparatus consisted of a SpectraSYSTEM P4000 pump system coupled to a highly sensitive UV6000LP diode array detector, equipped with 5 cm light path flow cell, setup for acquisition between 200 and 550 nm wavelengths (Thermo Fisher Scientific, Rodano, MI, Italy). Each compound included in chromatographic runs was identified and then quantified by comparing retention times, absorption spectra, and area of the peaks belonging to the chromatographic runs of mixtures composed by known concentrations of true ultrapure standard mixtures. Different acquisition wavelengths were used based on the different nature of the metabolites analyzed by HPLC. In particular, 260 nm was used for high energy phosphates and nicotinic coenzymes, 266 nm was used for malondialdehyde (MDA) (not detectable levels), while, 206 nm was used in the case of GSH and nitrite.

### 2.6 Statistical analysis

Graphpad Prism software (version 8.0) (Graphpad software, San Diego, CA, United States) was used to perform the statistical analysis. One-way analysis of variance (ANOVA), followed by Tukey’s *post hoc* test, was used for multiple comparisons. The statistical significance was set at *p*-values < 0.05. Data are reported as the mean ± SD of at least three samples.

## 3 Results

### 3.1 Carnosine protects microglia against the toxicity induced by LPS + ATP challenge

**FIGURE 1:**
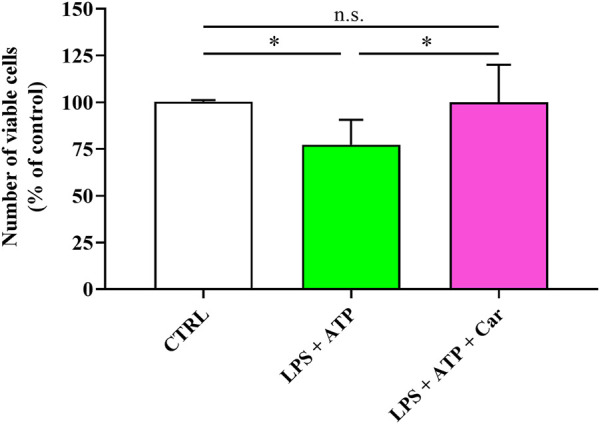
Number of viable cells in resting HMC3 and in HMC3 cells stimulated with LPS (100 ng/mL, 24 h) + ATP (5 mM, 30 min), in the absence or presence of carnosine (Car) (10 mM, 1 h pre-treatment). The number of viable cells is expressed as the percent variation with respect to the untreated (CTRL) cells. Values are means ± SD of four to five different cell counts. Standard deviations are represented by vertical bars. *significantly different, *p* < 0.05.

Before monitoring the potential protective effects of carnosine, we first investigated the effects of LPS + ATP on human microglia cells viability. The data reported in Figure 1 clearly show that the treatment of HMC3 cells with LPS + ATP significantly decreased the number of viable cells compared to untreated HMC3 cells (*p* < 0.05)**.** Despite the treatment with LPS + ATP, the pre-treatment with carnosine at the concentration of 10 mM was able to rescue the number of viable cells in HMC3 (*p* < 0.05 vs. LPS + ATP), giving cell number counts similar to that observed for untreated HMC3 cells.

### 3.2 Carnosine prevents the increase in intracellular ROS induced by LPS + ATP in HMC3 cells

**FIGURE 2:**
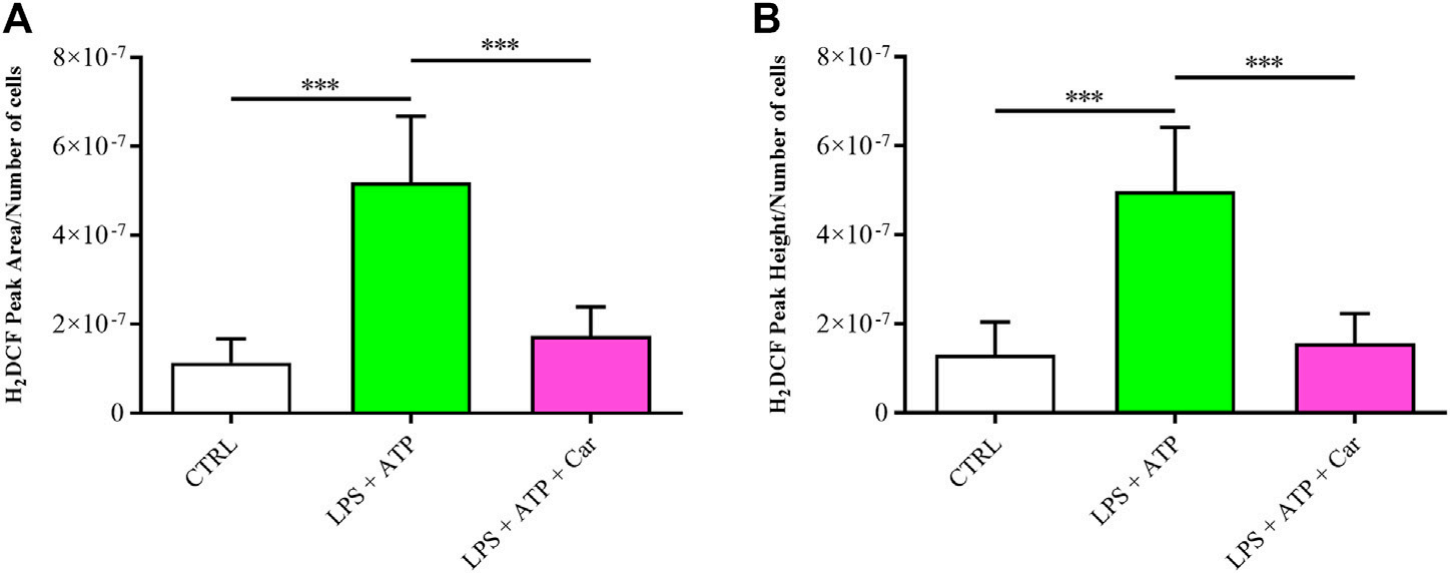
Detection of intracellular concentrations of ROS, expressed as **(A)** average peak area/number of cells or **(B)** average peak height/number of cells in resting HMC3 cells and in HMC3 cells challenged with LPS (100 ng/mL, 24 h) + ATP (5 mM, 30 min), in the absence or presence of carnosine (Car) (10 mM, 1 h pre-treatment). Values are means ± SD of three to four different samples. Each peak value was divided by the number of cells measured for the specific treatment. Standard deviations are represented by vertical bars. ***significantly different, *p* < 0.001.

As reported in Figure 2, the treatment of HMC3 with LPS + ATP was able to significantly increase the intracellular concentration of ROS compared to that observed in control cells (*p* < 0.001). The pre-treatment (1 h) of HMC3 cells with carnosine at the concentration of 10 mM was able to significantly (*p* < 0.001) decrease intracellular ROS levels despite the stimulation of cells with LPS + ATP.

### 3.3 Carnosine rescues cellular energy metabolism status in HMC3 cells challenged with LPS + ATP

**FIGURE 3:**
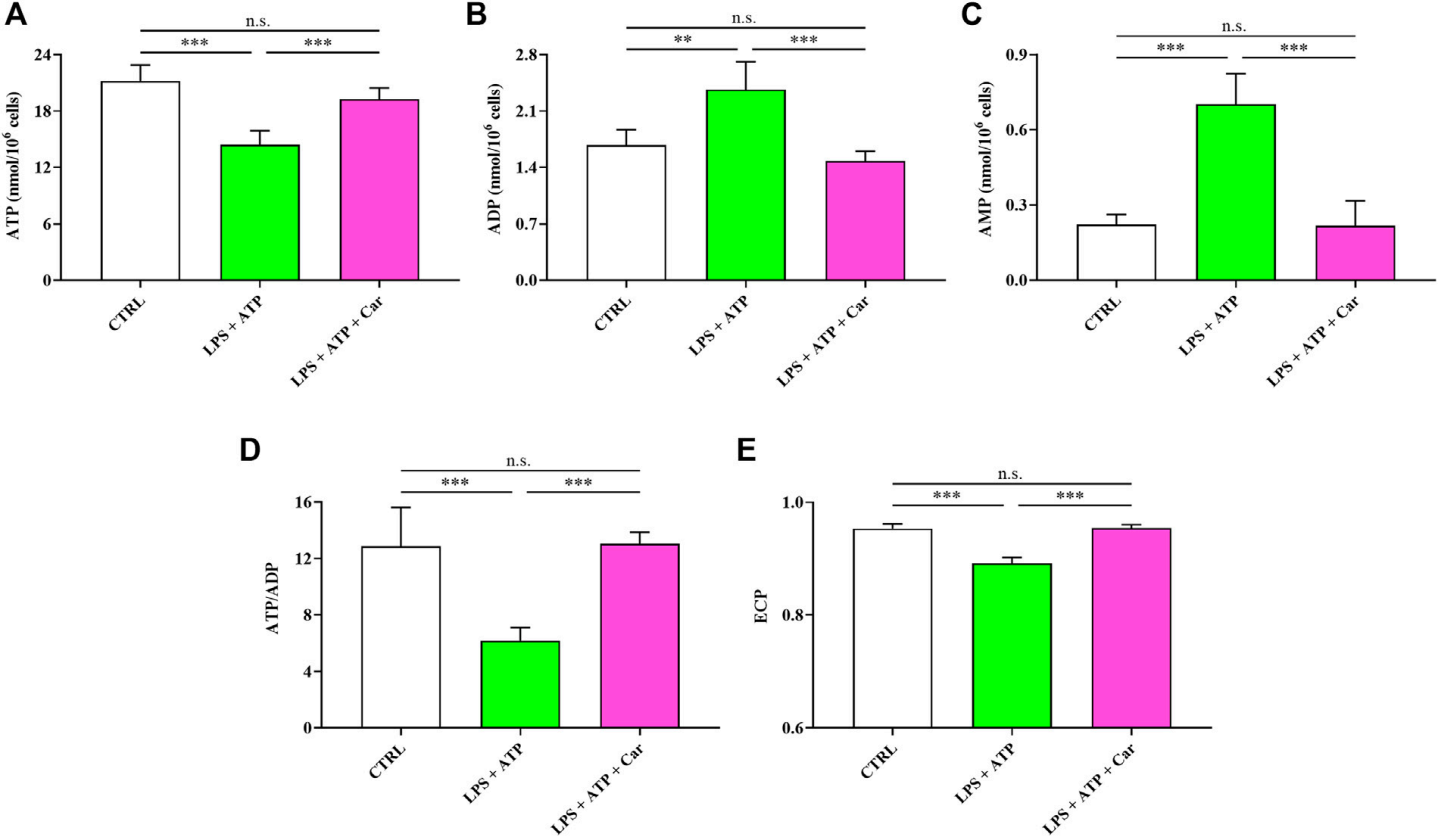
Values of **(A)** ATP, **(B)** ADP, **(C)** AMP, **(D)** ATP/ADP ratio, and **(E)** Energy Charge Potential (ECP = ATP +1/2ADP/ATP + ADP + AMP) determined in resting HMC3 cells and HMC3 cells exposed to LPS (100 ng/mL, 24 h) + ATP (5 mM, 30 min), in the absence or presence of carnosine (Car) (10 mM, 1 h pre-treatment). Data represent mean of four different samples. Standard deviations are represented by vertical bars. **Significantly different, *p* < 0.01; ***Significantly different, *p* < 0.001.

As shown in Figure 3, challenge of human microglia with LPS + ATP caused a deep imbalance in the cell energetic, as demonstrated by the 32% decline in the ATP concentration (*p* < 0.001 vs. CTRL) and the 42% and 218% increases in ADP (*p* < 0.01 vs. CTRL) and AMP (*p* < 0.001 vs. CTRL) levels, respectively. LPS + ATP challenge caused a 52% decrease in the ATP/ADP ratio (*p* < 0.001 vs. CTRL), strongly suggesting a strong decrease of the mitochondrial phosphorylating capacity deeply affecting the overall cell energy wellness, as indicated by the decline in the ECP determined in these cells (*p* < 0.001 vs. CTRL). Treatment with carnosine, under these stressing conditions, was able to rescue all the aforementioned parameters, allowing cells to have values of these indicators of the cell energy state significantly higher than those measured in LPS + ATP cells and, more relevant, not significantly different from those measured in controls.

**FIGURE 4:**
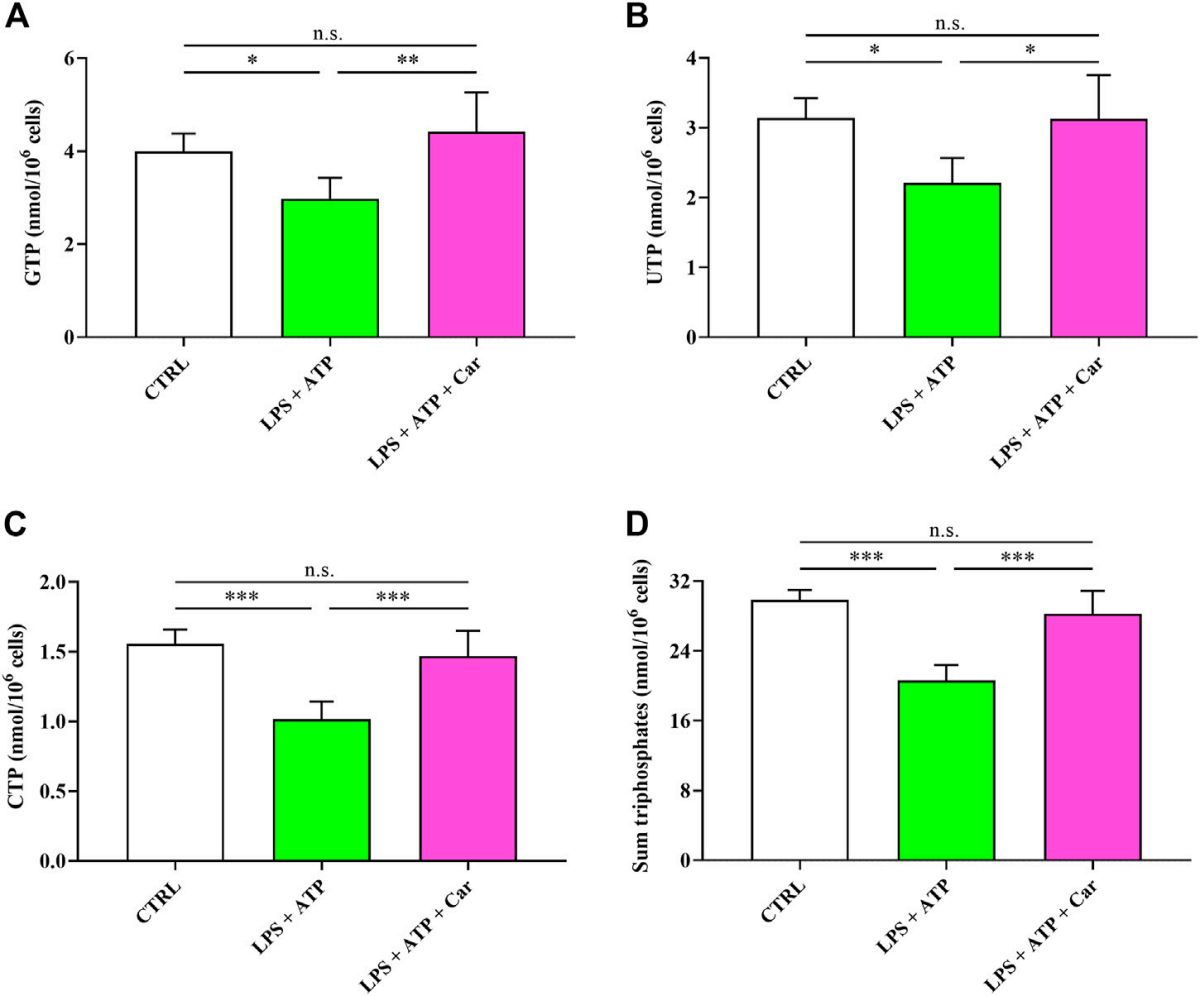
Values of **(A)** GTP, **(B)** UTP, **(C)** CTP, and **(D)** sum of triphosphates (ATP + GTP + UTP + CTP) determined in resting HMC3 cells and HMC3 cells exposed to LPS (100 ng/mL, 24 h) + ATP (5 mM, 30 min), in the absence or presence of carnosine (Car) (10 mM, 1 h pre-treatment). Data represent mean of four different samples. Standard deviations are represented by vertical bars. *Significantly different, *p* < 0.05; **Significantly different, *p* < 0.01; ***Significantly different, *p* < 0.001.

The energy crisis induced by LPS + ATP challenge negatively affected the concentrations of the other triphosphate nucleosides (GTP, UTP, and CTP) of great biochemical relevance in numerous fundamental cell processes (Figure 4). Notably, the sum of all triphosphate nucleosides (ATP, GTP, UTP, and CTP) was decreased by 31% in LPS + ATP-treated HMC3 cells. The beneficial effects of carnosine allowed to rescue the concentrations of these compounds to levels significantly higher than those measured in LPS + ATP cells and, again, not significantly different from those measured in controls.

**FIGURE 5:**
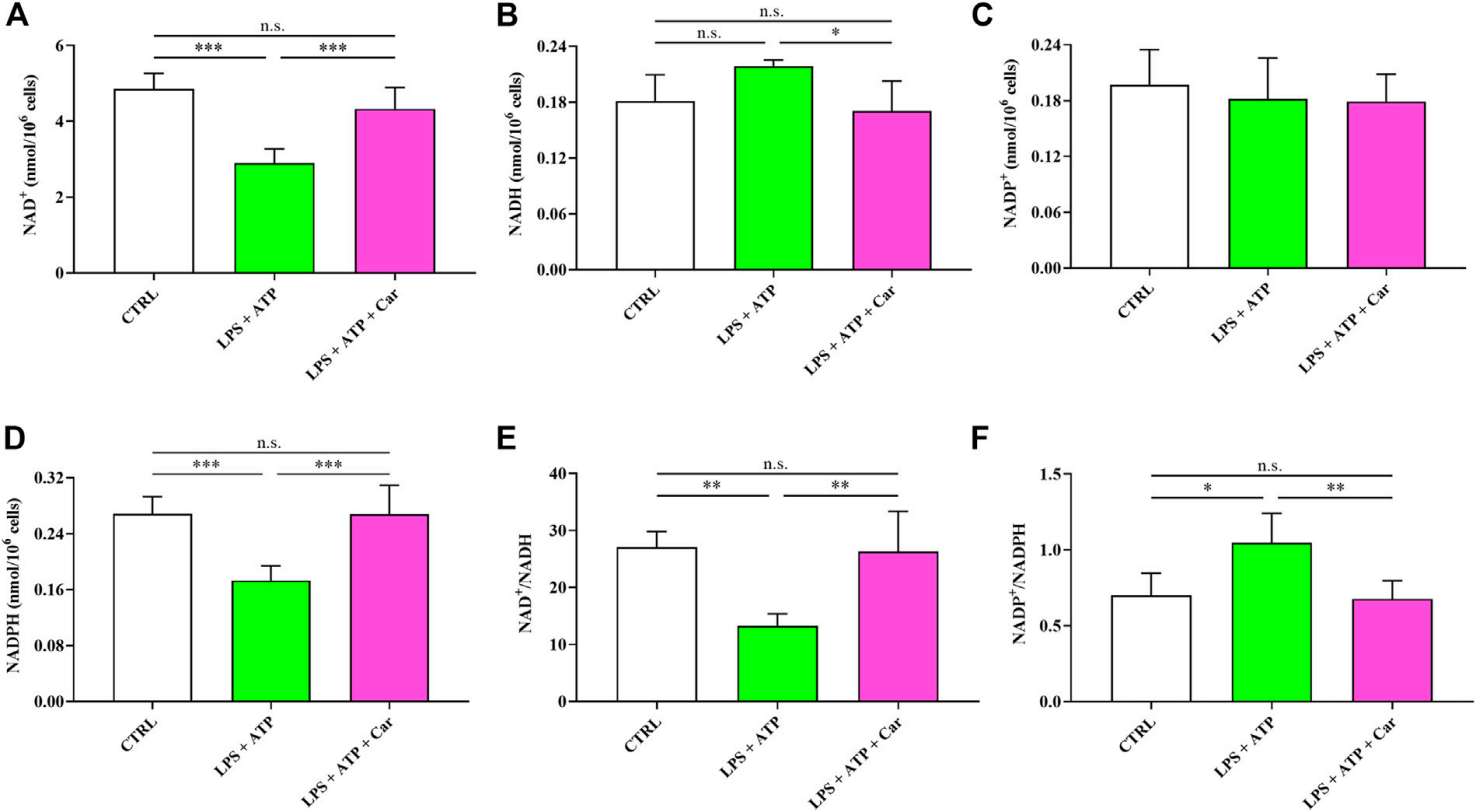
Values of oxidized **(A, C)** (NAD^+^ and NADP^+^) and reduced **(B, D)** (NADH and NADPH) nicotinic coenzymes determined in resting HMC3 cells and HMC3 cells exposed to LPS (100 ng/mL, 24 h) + ATP (5 mM, 30 min), in the absence or presence of carnosine (Car) (10 mM, 1 h pre-treatment). The oxidized/reduced ratios **(E, F)** are also shown. Data represent mean of four different samples. Standard deviations are represented by vertical bars. *Significantly different, *p* < 0.05; **Significantly different, *p* < 0.01; ***Significantly different, *p* < 0.001.

The negative effects of the exposure to LPS + ATP combined stimuli of human microglia on the concentrations and redox balance of nicotinic coenzymes is shown in Figure 5. Whilst NAD^+^ concentration underwent a 40% decrease (*p* < 0.001 vs. CTRL), no changes were observed in the case of NADP^+^. Concomitantly, NADH levels, although not significantly different from naïve cells, increased by 21% and those of NADPH decreased by 36% (*p* < 0.001 vs. CTRL). Consequently to the changes in the levels of oxidized and reduced forms of nicotinic coenzymes, both the NAD^+^/NADH as well as the NADP^+^/NADPH ratios showed significant changes, with the former undergoing a 51% decrease (*p* < 0.01 vs. CTRL) and the latter a 50% increase (*p* < 0.05 vs. CTRL). Impressively, the presence in the culture medium of carnosine was able to repristinate nicotinic coenzymes homeostasis, normalizing either the concentrations of oxidized and reduced forms of each coenzyme or the oxidized/reduced ratios.

**FIGURE 6:**
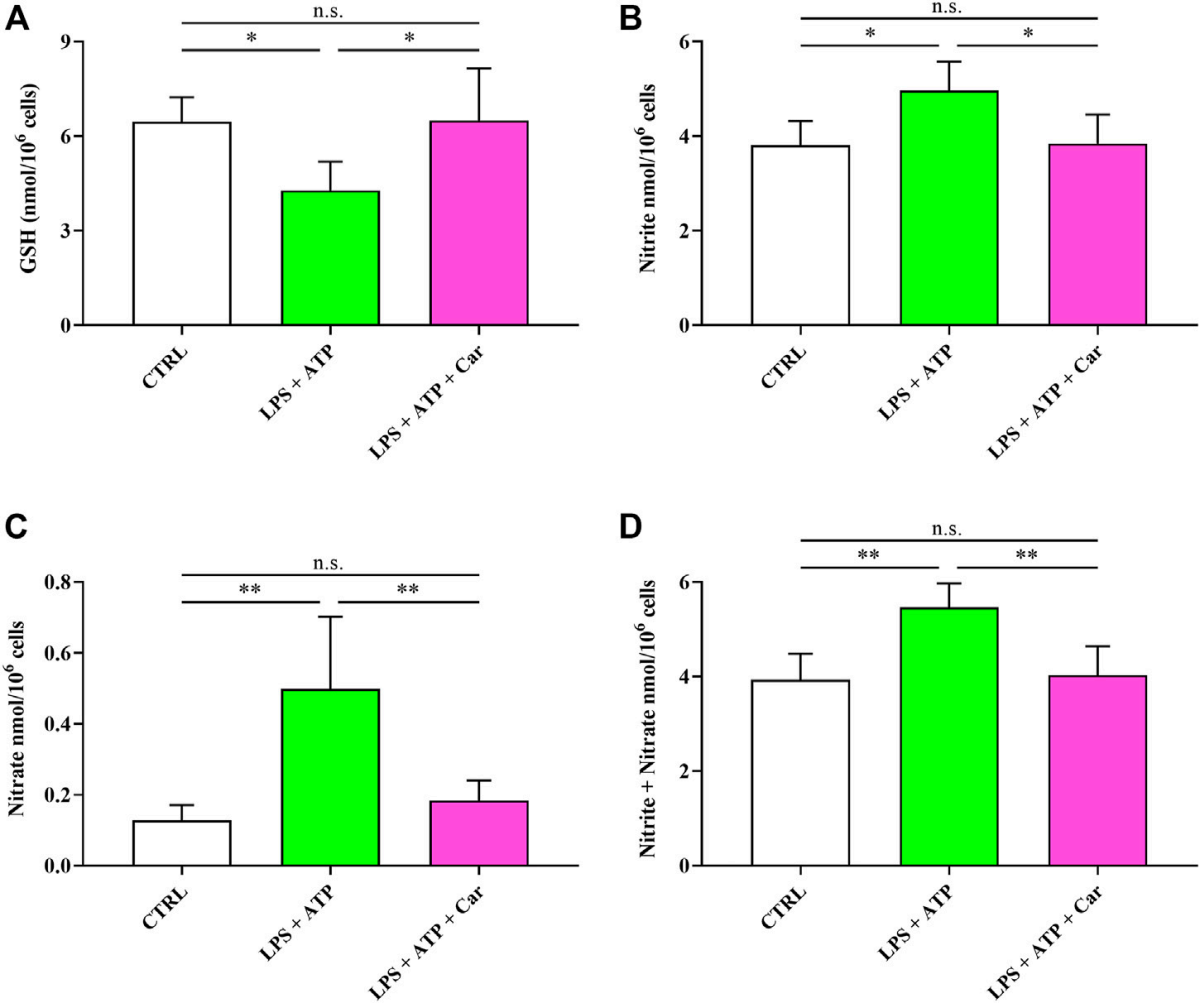
Values of **(A)** GSH, **(B)** nitrite, **(C)** nitrate, and **(D)** nitrite + nitrate determined in resting HMC3 cells and HMC3 cells exposed to LPS (100 ng/mL, 24 h) + ATP (5 mM, 30 min), in the absence or presence of carnosine (Car) (10 mM, 1 h pre-treatment). Data represent mean of four different samples. Standard deviations are represented by vertical bars. *Significantly different, *p* < 0.05; **Significantly different, *p* < 0.01.

Results illustrated in Figure 6 indicate that LPS + ATP causes a remarkable decrease in the concentration of GSH (−34%, *p* < 0.05), the main water-soluble antioxidant. The pro-inflammatory stimulus induced by LPS + ATP challenge also caused sustained overproduction of the stable end-products of NO catabolism, i.e., nitrite (+31%, *p* < 0.05) and nitrate (+289%, *p* < 0.001), therefore culminating in a condition of nitrosative stress. Certainly linked to the effects on cell energetic and nicotinic coenzyme homeostasis is the effect occurring to human microglia exposed to LPS + ATP + carnosine. Under these conditions, no differences with resting controls in any of the aforementioned parameters was observed, confirming the previous observations indicating that carnosine is capable to preserve cellular GSH content and prevent the increase in NO levels.

**FIGURE 7:**
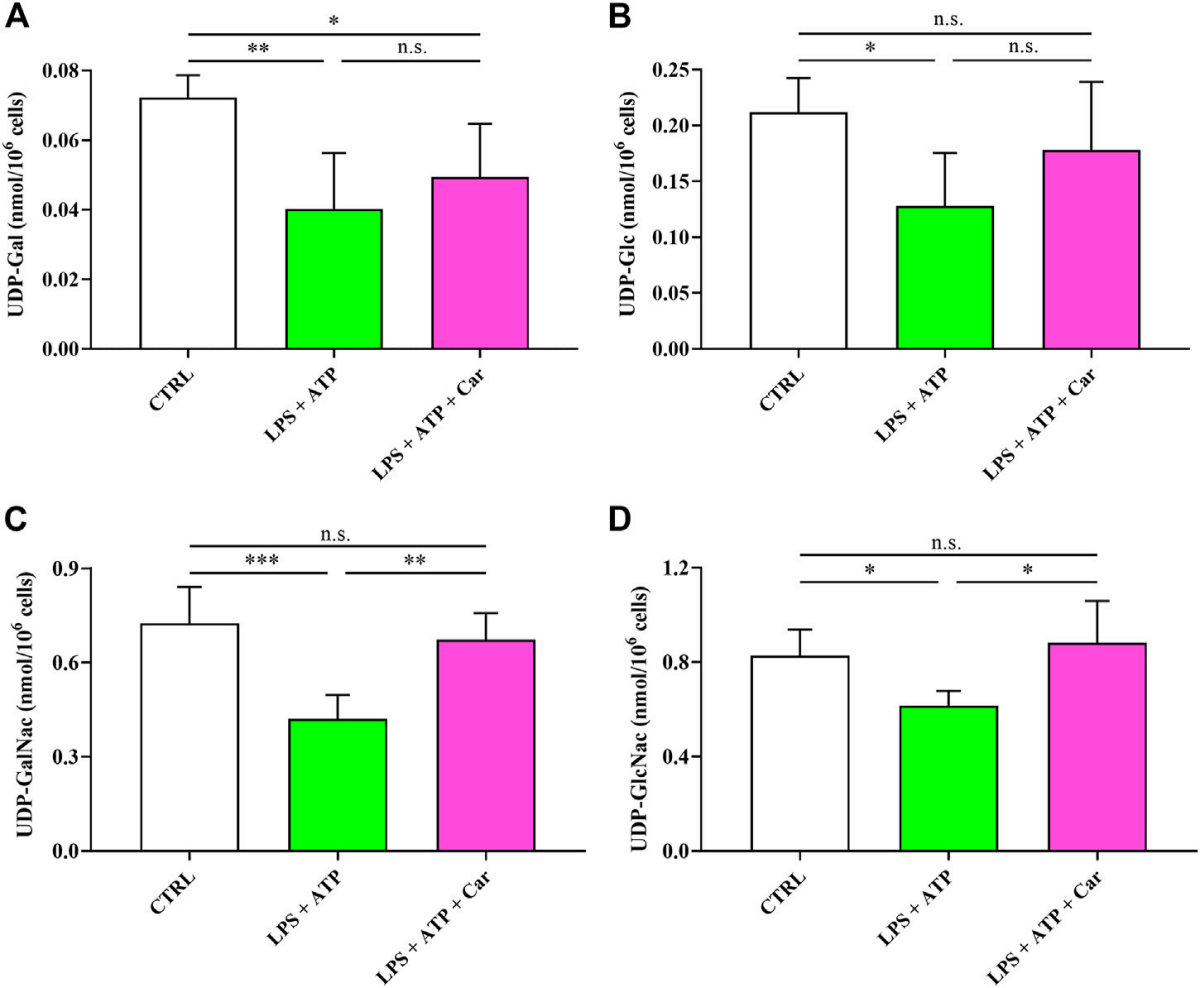
Values of **(A)** UDP-Gal, **(B)** UDP-Glc, **(C)** UDP-GalNac, and **(D)** UDP-GlcNac in resting HMC3 cells and HMC3 cells exposed to LPS (100 ng/mL, 24 h) + ATP (5 mM, 30 min), in the absence or presence of carnosine (Car) (10 mM, 1 h pre-treatment). Data represent mean of four different samples. Standard deviations are represented by vertical bars. UDP-Gal = UDP-galactose; UDP-Glc = UDP-glucose; UDP-GalNac = UDP-N-acetylgalactosamine; UDP-GlcNac = UDP-N-acetylglucosamine. *Significantly different, *p* < 0.05; **Significantly different, *p* < 0.01; ***Significantly different, *p* < 0.001.

Figure 7 shows that LPS + ATP negatively influences UDP-derivatives (UDP-Gal, UDP-Glc, UDP-GalNac, and UDP-GlcNac) ensuring the correct process of protein glycosylation indispensable for protein trafficking within and outside the cell. The addition of carnosine to LPS + ATP-treated HMC3 cells restored the concentrations of these UDP-derivatives but not that of UDP-Gal, the concentration of which was similar to that measured in cells challenged with LPS + ATP and lower than that measured in naïve cells (*p* < 0.05).

## 4 Discussion

Today, it is well-accepted in research community that microglia, myeloid cells and primary component of the brain immune system, play key roles in the regulation of numerous physiological processes that includes the trophic support of proliferation, survival, and differentiation of neural and other glial progenitor cells (Rodríguez et al., 2022) as well as synaptic plasticity (Yirmiya et al., 2015). As already stated, the deviation from microglial homeostasis can lead to different pathological conditions including neurodegenerative diseases (Liu and Hong, 2003; Glass et al., 2010; Stephenson et al., 2018) reason why the identification of the molecular mechanisms related to its pro-inflammatory and pro-oxidant activation are of great importance to develop therapeutic strategies (Dello Russo et al., 2017; Du et al., 2017). The activation of microglia can be considered as a mandatory path in the early phase of cognitive decline in dementia (Dhananjayan et al., 2017). Therefore in drug discovery processes, it becomes essential to identify pharmacological targets related to microglia activation to develop innovative therapeutic strategies for the treatment of cognitve disorders.

Among its numerous pharmacological activities, carnosine has shown the ability to regulate the activity of immune cells including microglia, enhancing their antioxidant capacity, increasing the expression and release of anti-inflammatory mediators and neurotrophic factors, and ameliorating the cellular energy metabolism of these cells (Caruso et al., 2017a; Fresta et al., 2017; Caruso et al., 2019b).

LPS-induced sickness behavior have been reported in rodents and humans (Lasselin et al., 2020). Numerous studies have also shown a strong association between inflammation and depression, with basal and LPS-stimulated inflammatory markers being associated with sickness behavior symptoms including anhedonia, low energy, and irritability (van Eeden et al., 2020). Furthermore, recent studies have suggested that depression can be regarded as a microglial disease (Wang et al., 2022) and LPS represents a well-validated tool to explore in human microglial cells the molecular mechanisms underlying affective and cognitive symptoms in depression (Kotulla et al., 2018).

Along this line, it has recently been demonstrated as the challenge of human HMC3 microglial cells with a combination of stimuli consisting of LPS + ATP is able to promote cell death due to the upregulation of pro-inflammatory genes (i.e., IL-1β and IL-18) and the activation of c-Fos/NLRP3/caspase-1 cascades (Li et al., 2018).

According to this scenario, in the present study, we first evaluated the modulation of the toxic effects induced by LPS + ATP challenge on HMC3 microglial cells in presence of carnosine dipeptide. When monitoring the number of viable cells under our experimental conditions, as expected, we observed that the treatment with LPS + ATP significantly decreased microglia viability, while the presence of carnosine prevented these toxic effects (Figure 1). We hypothesized that these protective effects could be related to the ability of carnosine to counteract oxidative stress in immune cells including microglia (Caruso et al., 2019b; Fresta et al., 2020). Based on this hypothesis, we investigated the correlation between the toxicity induced by LPS + ATP and intracellular ROS levels, well-known contributors to inflammatory and neurodegenerative phenomena (Togo et al., 2004; Massaad et al., 2009). The levels of intracellular ROS significantly increased following the challenge of HMC3 with LPS + ATP (Figure 2). This inductive effect in terms of ROS production is in accordance with numerous studies showing increased concentrations of different types of ROS in immune cells stimulated with LPS, alone or in combination with other pro-inflammatory stimuli such as interferon-γ (Hsu and Wen, 2002; Park et al., 2015). ROS levels were instead significantly decreased in the presence of carnosine (Figure 2). These results are in line with the well-recognized antioxidant activity of carnosine being associated with its ability to directly interact with these species (Klebanov et al., 1997) and with the presence of the imidazole ring of histidine part of its molecular structure (Caruso et al., 2017a). The results underlining the efficacy of carnosine in decreasing the levels of species related to oxidative stress are in line with other research studies in which carnosine exerted neuroprotection against oxidative stress *via* mitogen-activated protein kinase (MAPK) pathway modulation (Kulebyakin et al., 2012) or protected rat cerebellar cells against free radicals-induced damage (Lopachev et al., 2016).

The protective activity exerted by carnosine on HMC3, preventing LPS + ATP-induced cell death, could also depend on its ability to activate glial cells within the brain, stimulating both synthesis and release of neurotrophins, including brain-derived neurotrophic factor and nerve growth factor (Yamashita et al., 2018). In BV-2, an established experimental model to mimick neuroinflammation in primary microglia (Henn et al., 2009), carnosine was able to prevent cell death in cells challenged with Aβ oligomers through multiple mechanisms that included the rescue of IL-10 levels and the increase of the expression and the release of transforming growth factor beta-1 (Caruso et al., 2019c).

The challenge of human microglia with LPS + ATP caused a deep imbalance in the cell energetic (e.g., ATP↓, ADP↑, AMP↑, and ↓ATP/ADP ratio) (Figure 3) strongly suggesting a dramatic decrease of the mitochondrial phosphorylating capacity (Maldonado and Lemasters, 2014) deeply affecting the overall cell energy wellness, as also sustained by ECP decline. The energy crisis induced by LPS + ATP negatively affected the concentrations of GTP, UTP, and CTP (Figure 4), indicating profound alterations of metabolic pathways and cycles devoted to the cell energy supply. In agreement with previous studies, showing the ability of carnosine to ameliorate macrophage energy state (Caruso et al., 2019b; Fresta et al., 2020), the treatment with this dipeptide restored all the aforementioned parameters. With specific regards to HMC3 cells, it was recently demonstrated that carnosine leads to a generalized amelioration of the cell energy state, evaluated through the increase both in the ATP/ADP ratio and the ECP (Caruso et al., 2023). This suggests that the amelioration observed in the presence of carnosine could be the consequence of the ability of this dipeptide to counteract the deleterious effects of LPS + ATP challenge coupled to its capacity to enhance the basal cellular energy metabolism status.

LPS + ATP challenge also led to an unmbalance of redox nicotinic coenzymes (Figure 5). In particular, NAD^+^/NADH ratio was decreased, while NADP^+^/NADPH ratio was increased, in accordance with previous studies in which LPS led to significant changes in energy metabolism and mitochondrial functions in macrophages (Lee et al., 2019; Vijayan et al., 2019). Our data strongly suggest that LPS + ATP-treated cells appealed to glycolysis to counteract the energy crisis due to mitochondrial malfunctioning (decrease of the NAD^+^/NADH ratio), comprising both biosynthetic reactions and antioxidant defenses (increase of NADP^+^/NADPH ratio) (Amorini et al., 2016; Tataranni et al., 2017). Carnosine was able to repristinate nicotinic coenzymes homeostasis, normalizing either the concentrations of oxidized and reduced forms of each coenzyme or the oxidized/reduced ratios. Of course, this effect was strictly linked to the better cell energetic determined in LPS + ATP + carnosine treated cells, since the correct nicotinic coenzyme homeostasis is a prerequisite to support cell energy demand *via* electron transfer chain (ETC) coupled to oxidative phosphorylation (OxPHOS) and to allow the biosynthesis of structural and functional components (membrane lipids and nucleotides) and cell antioxidants (GSH) (Ghosh et al., 2018; Bernier et al., 2020; Cheng et al., 2021). These results are in line with previous studies showing that carnosine has beneficial effects on energy metabolism during periods of cell sufferance induced by various stimuli (Macedo et al., 2016; Ouyang et al., 2016).

The remarkable decrease in GSH concentration (Figure 6), representing the main water-soluble antioxidant (Lazzarino et al., 2019), is certainly connected to the altered levels of NADP^+^, NADPH, and NADP^+^/NADPH ratio leading to diminished cell capacity to reduce the increase in oxidized GSH under conditions of increased oxidative stress. Interestingly our experimental model of neuroinflammation mimicks what observed in patients with major depressive disorder (MDD), where a significant reduction of GSH levels has been detected in the brain (Gawryluk et al., 2011). Further studies are needed to understand whether the clinical efficacy of carnosine as add-on treatment in MDD (Araminia et al., 2020) might be related to its antioxidant activity and its rescuing effects on GSH levels.

The pro-inflammatory stimulus induced by LPS + ATP also caused sustained overproduction of nitrite and nitrate, therefore culminating in a condition of nitrosative stress (May et al., 2004; Ergün et al., 2011). Carnosine not only restored the levels of GSH, in accordance to a recent study by Jamshidzadeh *et al.* (Jamshidzadeh et al., 2017), but was also able to reduce nitrite and nitrate to their initial levels, confirming, also in human microglia, its antioxidant properties previously showed in murine models (Caruso et al., 2019c; Solana-Manrique et al., 2022).

As reported in Figure 7, LPS + ATP negatively influences UDP-derivatives (UDP-Gal, UDP-Glc, UDP-GalNac, and UDP-GlcNac), representing key mediators in ensuring the correct process of protein glycosylation indispensable for protein trafficking within and outside the cell (Akella et al., 2019; Giallongo et al., 2020). In this, the correct functioning of the hexosamine biosynthetic pathway (HBP), strictly dependent on adequate UTP availability, is fundamental for the maintenance of the correct levels of the aforementioned UDP-derivatives. HBP and protein glycosylation are associated to the correct functioning of endoplasmic reticulum (ER) (Srinivasan et al., 2009). Stressors altering HBP functionality, in our conditions certainly because of UTP depletion, lead to ER stress and modification in the correct functioning of the complex process of protein glycosylation. The addition of carnosine to LPS + ATP-treated cells restored the concentrations of these UDP-derivatives (except UDP-Gal). It is however possible to affirm that the presence of carnosine, by restoring the concentrations of 3 of the 4 UDP-derivatives, generated highly favorable conditions for a correct HBP and glycosylation process with reduction of stressing conditions of ER. It remains essential to evaluate and validate in future studies the same metabolic alterations in animal models of AD and depression, sharing behavioural and molecular alterations.

## 5 Conclusion

In the present study we were able to show for the first time that carnosine suppresses cell death induced by LPS + ATP combination in HMC3 microglia by decreasing oxidative stress measured as intracellular ROS, nitrite, and nitrate levels, also rescuing GSH content. The protective activity exerted by carnosine was also attributable to its ability to completely restore basal energy metabolism conditions as evidenced by the positive modulation of high-energy triphosphates, nicotinic coenzymes, and UDP-derivatives. Our results suggest a therapeutic potential of carnosine as a new pharmacological tool in the context of cognitive disorders, such as depression and AD, characterized by microglia over-activation, oxidative stress, and energy unbalance.

## Data Availability

The original contributions presented in the study are included in the article/Supplementary Material, further inquiries can be directed to the corresponding author.
