# Supplementary material for: Microfluidic/HPLC combination to study carnosine protective activity on challenged human microglia: Focus on oxidative stress and energy metabolism
Source: Front Pharmacol. 2023 Mar 29;14:1161794. doi: 10.3389/fphar.2023.1161794 (PMC10095171; doi:10.3389/fphar.2023.1161794)
Supplement: Supplementary file 1 [file DataSheet1.PDF]

Supplementary Material

Microfluidic/HPLC combination to study carnosine protective activity on challenged human microglia: focus on oxidative stress and energy metabolism

Anna Privitera[1,2,§], Vincenzo Cardaci[3,4,§], Dhanushka Weerasekara[5,6], Miriam Wissam Saab[2], Lidia Diolosà[1], Annamaria Fidilio[7], Renaud Blaise Jolivet[8], Giuseppe Lazzarino[2], Angela Maria Amorini[2], Massimo Camarda[9], Susan Marie Lunte[5,6,10], Filippo Caraci[1,7,†], Giuseppe Caruso[1,7,†,*]

*** Correspondence:** Corresponding Author: giuseppe.caruso2@unict.it

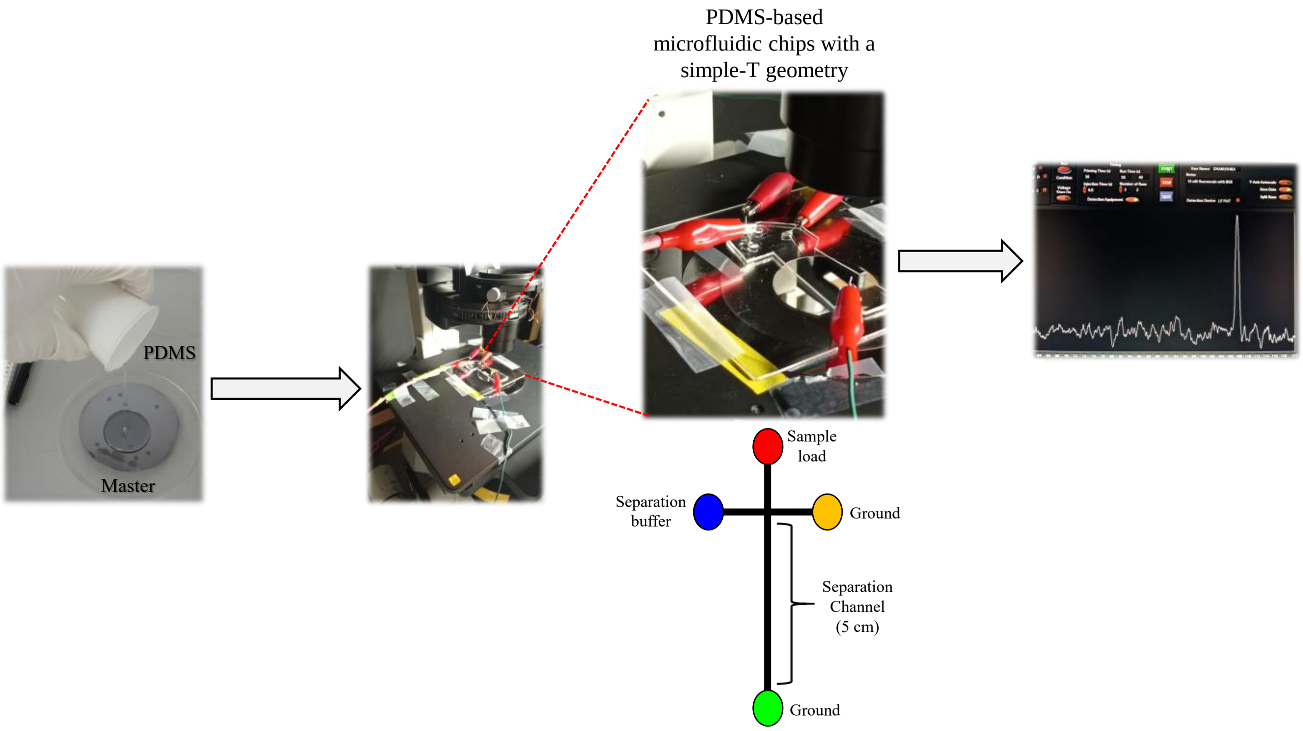

Supplementary Figure 1. Illustration of the PDMS-based microfluidic chips with a simple-T geometry made by mixing PDMS prepolymer and curing agent and used to analyze cell lysates.
